# Suitable Reference Genes for Accurate Gene Expression Analysis in Parsley (*Petroselinum crispum*) for Abiotic Stresses and Hormone Stimuli

**DOI:** 10.3389/fpls.2016.01481

**Published:** 2016-09-30

**Authors:** Meng-Yao Li, Xiong Song, Feng Wang, Ai-Sheng Xiong

**Affiliations:** State Key Laboratory of Crop Genetics and Germplasm Enhancement, College of Horticulture, Nanjing Agricultural UniversityNanjing, China

**Keywords:** parsley, reference gene, qPCR, abiotic stress, hormone stimuli

## Abstract

Parsley, one of the most important vegetables in the Apiaceae family, is widely used in the food, medicinal, and cosmetic industries. Recent studies on parsley mainly focus on its chemical composition, and further research involving the analysis of the plant's gene functions and expressions is required. qPCR is a powerful method for detecting very low quantities of target transcript levels and is widely used to study gene expression. To ensure the accuracy of results, a suitable reference gene is necessary for expression normalization. In this study, four software, namely geNorm, NormFinder, BestKeeper, and RefFinder were used to evaluate the expression stabilities of eight candidate reference genes of parsley (*GAPDH, ACTIN, eIF-4*α, *SAND, UBC, TIP41, EF-1*α, and *TUB*) under various conditions, including abiotic stresses (heat, cold, salt, and drought) and hormone stimuli treatments (GA, SA, MeJA, and ABA). Results showed that *EF-1*α and *TUB* were the most stable genes for abiotic stresses, whereas *EF-1*α, *GAPDH*, and *TUB* were the top three choices for hormone stimuli treatments. Moreover, *EF-1*α and *TUB* were the most stable reference genes among all tested samples, and *UBC* was the least stable one. Expression analysis of *PcDREB1* and *PcDREB2* further verified that the selected stable reference genes were suitable for gene expression normalization. This study can guide the selection of suitable reference genes in gene expression in parsley.

## Introduction

Gene expression analysis is an important tool from studying the complex biological processes, such as signal transduction, metabolic pathways, and plant development. In the qualitative or quantitative analysis of the expression of the sample or target gene, qPCR is the most effective method because of its simplicity, high sensitivity and specificity (Wong and Medrano, 2005). However, to date, universal reference gene in plants or animals has not been reported yet (Warrington et al., 2000; Schmittgen and Zakrajsek, 2000). Just borrowed the reference genes in other species might get the wrong expression model. Therefore, evaluation of the stability of reference genes is particularly important.

Housekeeping genes, such as *ACTIN, EF-1*α, and *UBQ*, are usually used as candidate reference genes because of their role in maintaining cell survival irrespective of different physiological conditions (Bustin, 2002). However, recent studies showed that the expression patterns of housekeeping genes also change under different experimental conditions (Thellin et al., 1999; Suzuki et al., 2000). In a study of carrot, *GAPDH* displayed the maximum stability for most of single abiotic stresses (Tian et al., 2015), whereas *GAPDH* appeared to be the least stable gene during the five developmental stages of the plant (Wang et al., 2016). In addition, the stably expressed reference gene in one species may not be suitable in other species (Andersen et al., 2004; Gutierrez et al., 2008). For example, *TUB-B, TUB-A*, and *UBC* are the most stable reference genes during celery development (Li et al., 2016), whereas in cherries, *TUB-A* is the least stable gene during fruit development (Ye et al., 2015).

Parsley (*Petroselinum crispum* L.), a member of the Apiaceae family, is a widely cultivated spice. Parsley is also reported as a medicinal plant because it is rich in antioxidants, essential oil, flavonoids, and vitamins (Suhaj, 2006; Zhang et al., 2006). Abiotic stresses, including extreme temperature, salinity, and drought, are the major factors affecting the growth and yield of parsley. The physiological function of the plant is affected by abiotic stresses, and the expression patterns of mRNA and protein also change accordingly (Li et al., 2014). Studies showed that most of the hormones can regulate plant physiological activities, and exogenous plant hormones can improve plant resistance to varying degrees (Eraslan et al., 2007; Bari and Jones, 2009). For example, ABA is called “stress hormone” in plants because it plays an important role in resisting salinity, drought, and low temperature (Narendra, 2007). Plants have established their own resistance strategies in response to extreme adverse conditions (Chen and Zhu, 2004; Katagiri, 2004). Research on the molecular biology of parsley can help in screening the stress resistant genes and accelerating the breeding process. One way of studying the function of stress-resistance genes is to detect their expression levels. However, no systematic study regarding the selection of suitable reference genes for qPCR normalization in parsley has been published to date.

In this study, eight candidate reference genes, including *GAPDH, ACTIN, eIF-4*α, *SAND, UBC, TIP41, EF-1*α, and *TUB*, were assessed by qPCR under various abiotic stresses (cold, heat, salt, and drought) and hormone stimuli treatments (GA, SA, MeJA, and ABA). The expression stabilities of these candidate genes were evaluated by four statistical tools, namely geNorm (Vandesompele et al., 2002), NormFinder (Andersen et al., 2004), Bestkeeper (Pfaffl et al., 2004), and RefFinder (Xie et al., 2012). Additionally, the target genes, *PcDREB1* and *PcDREB2*, were used to identify the best-ranked reference genes. The stable reference genes will enable the more accurate and reliable qPCR analysis of parsley.

## Materials and methods

### Plant materials and treatments

The curly parsley (*P. crispum* L. cv. Moxi) seeds were sown in plastic pots containing a soil/vermiculite mixture (3:1) in a controlled-environment growth chamber under a photoperiod of 16 h with 300 μmol m^−2^s^−1^ light intensity at 25°C and 8 h dark condition at 16°C. After 8 weeks, the healthy seedlings were used for different treatments. For cold and heat treatments, the seedlings were kept in a growth chamber under 4°C and 38°C for 2 h. For salt and drought treatments, the soils were irrigated with 0.2 M NaCl and 20% PEG6000, respectively. For hormone treatments, the leaves were sprayed with 1.4 mM GA, or 1.4 mM SA, or 0.8 mM MeJA, or 0.1 mM ABA (Jiang et al., 2014). Plants were irrigated or sprayed only once, with untreated plants used as control. All the plants were kept in the same growth conditions for 2 h, and then the leaf samples were harvested. Three biological replicates were performed in different pots for each treatment. All the materials were immediately frozen in liquid nitrogen and then stored at −80°C.

### RNA isolation and cDNA synthesis

Total RNA was extracted from the samples using the RNA simple Total RNA Kit (Tiangen, Beijing, China), and genomic DNA was removed by RNase-free DNase I (Takara, Dalian, China). The quantity and quality of RNA samples were measured by agarose gel electrophoresis and Nanodrop ND 1000 spectrophotometer (Nanodrop Technologies Inc., Delaware, USA). RNA samples with an OD_260/280_ value between 1.8 and 2.2 were considered as high-purity RNA. A total of 1.0 μg RNA was reverse transcribed into cDNA using PrimeScript RT reagent Kit (Takara, Dalian, China).

### Primer design and qPCR analysis

Based on the transcriptome database of parsley built by our group (Lab of Apiaceae Plant Genetics and Germplasm Enhancement, Nanjing Agricultural University, Nanjing, China) (Li et al., 2014), eight candidate reference genes (*GAPDH, ACTIN, eIF-4*α, *SAND, UBC, TIP41, EF-1*α, and *TUB*) were selected as their corresponding homologs genes performed well in other plants in previous studies (Czechowski et al., 2005; Paolacci et al., 2009) and they were cloned in parsley. We submitted the nucleotide sequences to GenBank, and the corresponding accession numbers are KX784033 (*eIF-4*α), KX784034 (*ACTIN*), KX784035 (*TIP41*), KX784036 (*GAPDH*), KX784037 (*SAND*), KX784038 (*EF-1*α), KX784039 (*TUB*), and KX784040 (*UBC*).

The reverse transcribed cDNAs were diluted to ten-fold series (10, 10^2^, 10^3^, 10^4^, and 10^5^ X dilutions) for determination of the amplification efficiency (*E* %) of primers and correlation coefficient (*R*^2^), and 18-fold dilution was conducted for qPCR analysis. qPCR was performed using the MyiQ Single Color Real-Time PCR Detection System (Bio-rad, Hercules, CA, USA). The reaction mixture with a total volume of 20 μL contained 2 μL diluted cDNA, 0.4 μL of each 10 μM primer, 7.2 μL ddH2O, and 10 μL SYBR Green I mix (Takara, Dalian, China). The amplification program was 95°C for 30 s, followed by 40 cycles at 94°C for 5 s, and 60°C for 30 s. After amplification, a melting curve (65–95°C with at increments of 0.5°C) was generated for each reaction to verify the specific amplification. A no-template control (cDNAs were replaced with sterilized water) was included in each run for each gene to confirm the absence of nonspecific products. Each PCR reaction was repeated thrice with three biological replicates. Primers for the eight genes were designed by Primer Premier 6 with the following parameters: 57–63°C annealing temperature, 18–22 bp primer length, 40–60% GC contents, and 90–300 bp amplicon length. The primer sequences are listed in Table 1.

**Table 1:** **Details of candidate reference genes and primers used in qPCR**.

**Gene**	**Primer sequence (5′–3′) forward/reverse**	**Amplicon length (bp)**	**PCR efficiency (*E* %)**	**Correlation coefficient (*R*^2^)**	**Tm (°C)**
*ACTIN*	CTGGATTCTGGTGATGGTGTGA/CTCAGCAGTGGTGGTGAACAT	159	106.0	0.997	82.5
*EF-1α*	AGGCTCTTCAGGAGGCTCTTC/CAATGTGACAGGTGTGGCAATC	216	95.2	0.992	83.5
*eIF-4α*	CACGGAGACATGGATCAGAACAC/GAGACCTGCTGGACATCAATACC	119	95.2	0.993	81.5
*GAPDH*	TCGGACGCATTGGCAGGAA/GCTGGAGTGGATCTCTGTTGGA	228	96.5	0.990	83.5
*TIP41*	GGAGGACTGTGAGGAACGAATTG/TAAGCACGCCATCAACTCTAAGC	194	108.6	0.992	81.5
*TUB*	ATGGTTCTTGACAATGAGGCACTA/GCTTCCGCAGATCCGAGTTG	160	101.3	0.994	84.0
*SAND*	CAGATGCCGTGTTCTCTTCTCT/CACCAACCAGACTGATGACCTT	192	105.7	0.993	83.0
*UBC*	ATGGCGAATAGCAGCAATCTCC/TGTTATCTTCAGACGGCGATGC	103	101.2	0.993	83.5

### Data analysis

The Cq value (quantification cycle) of each reference gene under different conditions was recorded using the qPCR system. Four statistical tools were used to rank to expression stability of the reference genes: geNorm (Vandesompele et al., 2002), NormFinder (Andersen et al., 2004), Bestkeeper (Pfaffl et al., 2004), and RefFinder (Xie et al., 2012). For geNorm and NormFinder, the raw Cq values were converted into the relative quantity values *via* the formula, 2^−ΔCq^. geNorm ranks the gene expression stability (*M*-value) based on the pairwise variation (V) for the gene compared with all other reference genes. The default limit of *M*-value is 1.5, and the lowest *M*-value indicates the most stable gene. geNorm also calculates the pairwise variation (V_n/n+1_) between normalization factors (NFn and NFn+1, *n* ≥ 2) to determine the optimal number of reference genes in qPCR normalization. With a V_n/n+1_ below 0.15, introducing an additional reference gene is not necessary. NormFinder generates a similar measure through estimated intragroup and intergroup expression variations and combines the variation into a stability value for each gene. BestKeeper ranks the stability based on the calculation of the standard deviation (SD) and the coefficient of variance (CV) with the Cq values. The reference gene with the lowest SD- and CV-values is identified as the most stable gene. RefFinder is a web-based tool that integrates the four computational programs (geNorm, Normfinder, BestKeeper, and the comparative delta-Ct method) to comprehensively rank the tested candidate reference genes.

### Assessment of normalization by qPCR

qPCR was also performed to analyze the expression levels of the target genes, *PcDREB*1and *PcDREB*2. The relative expression levels were calculated with the 2^−ΔΔCT^ method (Pfaffl, 2001). The qPCR primer pairs for *PcDREB*1 were 5′-ACTCGGATCTGGCTAGGCACAT-3′ and 5′-TGGAGAAGCGGTCACCTCATC A-3′, and for *PcDREB2*, the primer paris were 5′-TTCCAGAACTCGCTCACCACT T-3′ and 5′- GACTCGCTCCATTCGTAACTCG-3′.

## Results

### Primer specificity and efficiency of candidate reference genes

**Figure 1:**
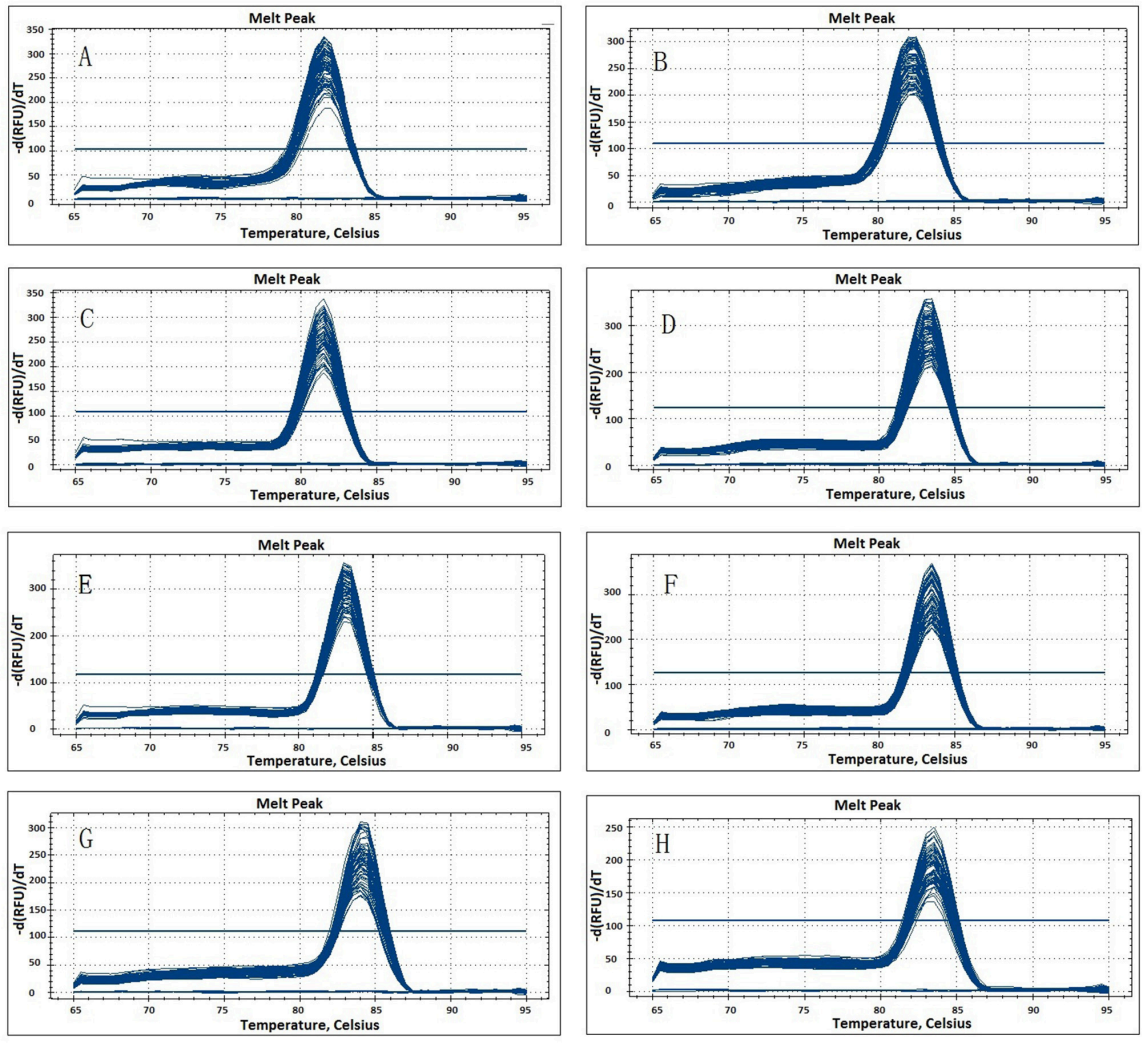
**Melting curves generated for eight candidate reference genes by qPCR in parsley. (A)** *eIF-4*α; **(B)** *ACTIN*; **(C)** *TIP41*; **(D)** *GAPDH*; (**E)** *SAND*; **(F)** *EF-1*α; **(G)** *TUB*; **(H)** *UBC*.

The eight candidate reference genes (*GAPDH, ACTIN, eIF-4*α, *SAND, UBC, TIP41, EF-1*α, and *TUB*) were selected from the transcriptome database, and they were chosen as their corresponding homologs genes performed well in qPCR normalization. The performances of all primer pairs were tested by standard curve analysis. The amplification efficiencies (*E*%) ranged from 95.2% to 108.6% and correlation coefficient (*R*^2^) ranged from 0.990 to 0.997 (Table 1, Figure S1), the results agreed with the standard (Ramakers et al., 2003). A single peak from all samples in the melting curve analysis showed that the primers were displayed without non-specific amplification (Figure 1).

### Cq value analysis

**Figure 2:**
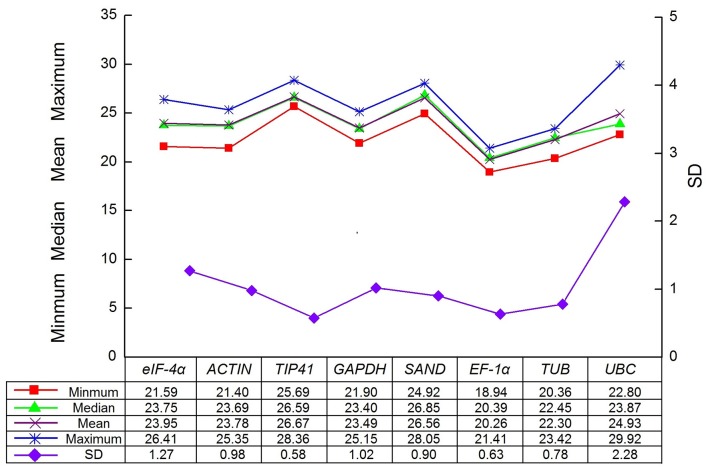
**Data statistics of Cq values of candidate reference genes in parsley**.

The expression levels of the eight candidate reference genes (*GAPDH, ACTIN, eIF-4*α, *SAND, UBC, TIP41, EF-1*α, and *TUB*) were presented as Cq values. To further understand of the Cq values, a distribution diagram was drawn (Figure 2). The Cq values of eight reference genes presented a relatively wide range from 18.94 for *EF-1*α to 29.92 for *UBC* in all tested samples. A low Cq value represents high expression level, which indicates that the gene has low expression. In our study, *EF-1*α was the most abundant gene with the lowest mean Cq value (20.26), whereas *TIP41* was the least expressed gene with the highest mean Cq value (29.92). In addition, among all the samples, the SD of *TIP41* was the smallest (0.58), whereas UBC showed the maximum variability (*SD* = 2.28).

### Expression stability of candidate reference genes

In our study, each reference gene was subjected to eight experiment treatments. The eight treatment sets were analyzed individually. To achieve a more comprehensive analysis, these eight sets were divided into three groups for further analysis: “Abiotic stress” (heat, cold, salt, and drought), “Hormone stimuli” (SA, GA, ABA, and MeJA), and “Total” (composed of all the treatment sets). Four different software, namely geNorm, NormFinder, Bestkeeper, and RefFinder, were used to analyze the stability of candidate reference genes.

#### geNorm analysis

geNorm ranks reference genes by calculating the *M*-value. A candidate gene with the *M*-value below 1.5 is considered as a stably expressed gene, and a lower *M*-value indicates greater stability. The expression stability rankings based on the *M*-values are displayed in Tables 2, 3. The *M*-values for the tested genes in all samples and groups were all lower than 1.5. For each individual treatment (Table 2), *TUB* was the most stably expressed gene with the least *M*-value under heat, drought, and GA conditions, whereas *EF-1*α and *GAPDH* were the most stably expressed under salt, ABA, MeJA, and SA conditions. For cold treatment, *GAPDH* and *eIF-4*α were the most stable candidate genes. For the three groups (Table 3), *EF-1*α and *GAPDH* were the most stably expressed genes under “Hormone stimuli,” and *TUB* and *EF-1*α were the most stable genes under “Abiotic stress” and “Total.” Among all the groups, *eIF-4*α and *UBC* were the least stable genes.

**Table 2:** **Gene expression stability under individual stress conditions ranked by geNorm, NormFinder, and BestKeeper**.

**Treatments**	**Rank**	**geNorm**		**NormFinder**		**BestKeeper**			**RefFinder**	
		**Gene**	**Stability**	**Gene**	**Stability**	**Gene**	**SD**	**CV**	**Gene**	**Comprehensive ranking values**
Heat	1	*TUB*	0.25	*TIP41*	0.02	*GAPDH*	0.14	0.57	*EF-1α*	1.68
	2	*ACTIN*	0.25	*EF-1α*	0.02	*ACTIN*	0.69	3.01	*TUB*	2.63
	3	*eIF-4α*	0.48	*ACTIN*	0.07	*TUB*	0.71	2.99	*GAPDH*	3.22
	3	*EF-1α*	0.49	*SAND*	0.10	*eIF-4α*	1.22	5.29	*TIP41*	5.00
	5	*SAND*	0.58	*TUB*	0.17	*EF-1α*	1.25	5.88	*eIF-4α*	6.24
	6	*TIP41*	0.66	*eIF-4α*	0.17	*SAND*	1.42	5.02	*SAND*	5.96
	7	*GAPDH*	0.79	*GAPDH*	0.21	*TIP41*	1.80	6.32	*ACTIN*	6.74
	8	*UBC*	1.42	*UBC*	0.26	*UBC*	4.23	15.49	*UBC*	8.00
Cold	1	*GAPDH*	0.15	*TUB*	0.01	*eIF-4α*	0.30	1.26	*EF-1α*	1.57
	2	*eIF-4α*	0.15	*eIF-4α*	0.01	*GAPDH*	0.32	1.27	*GAPDH*	1.73
	3	*EF-1α*	0.42	*GAPDH*	0.02	*ACTIN*	0.35	1.48	*eIF-4α*	2.11
	3	*TUB*	0.44	*EF-1α*	0.02	*EF-1α*	0.80	3.69	*ACTIN*	3.94
	5	*UBC*	0.52	*SAND*	0.06	*TUB*	0.81	3.44	*TUB*	4.47
	6	*SAND*	0.65	*UBC*	0.06	*UBC*	1.06	3.49	*TIP41*	6.00
	7	*TIP41*	0.76	*ACTIN*	0.12	*SAND*	1.37	4.80	*UBC*	7.00
	8	*ACTIN*	0.90	*TIP41*	0.17	*TIP41*	1.69	5.92	*SAND*	8.00
Drought	1	*TUB*	0.21	*EF-1α*	0.02	*ACTIN*	0.17	0.74	*ACTIN*	1.86
	2	*GAPDH*	0.21	*GAPDH*	0.03	*eIF-4α*	0.34	1.41	*TUB*	1.97
	3	*EF-1α*	0.24	*TUB*	0.06	*EF-1α*	1.00	4.69	*GAPDH*	2.91
	3	*SAND*	0.53	*SAND*	0.09	*TUB*	1.06	4.50	*EF-1α*	3.31
	5	*TIP41*	0.65	*eIF-4α*	0.12	*GAPDH*	1.16	4.82	*eIF-4α*	3.31
	6	*eIF-4α*	0.79	*TIP41*	0.13	*SAND*	1.53	5.42	*TIP41*	5.18
	7	*ACTIN*	0.91	*ACTIN*	0.18	*TIP41*	1.89	6.66	*SAND*	7.00
	8	*UBC*	1.48	*UBC*	0.22	*UBC*	4.07	14.83	*UBC*	8.00
Salt	1	*EF-1α*	0.22	*TUB*	0.03	*ACTIN*	0.21	0.91	*EF-1α*	1.19
	2	*GAPDH*	0.22	*SAND*	0.04	*eIF-4α*	0.29	1.18	*ACTIN*	1.41
	3	*TUB*	0.31	*UBC*	0.06	*TUB*	1.24	5.33	*UBC*	3.22
	3	*SAND*	0.48	*EF-1α*	0.11	*EF-1α*	1.45	6.90	*GAPDH*	3.94
	5	*TIP41*	0.59	*GAPDH*	0.20	*GAPDH*	1.52	6.41	*TUB*	4.73
	6	*eIF-4α*	1.02	*eIF-4α*	0.22	*SAND*	1.83	6.55	*SAND*	6.00
	7	*ACTIN*	1.20	*ACTIN*	0.24	*TIP41*	2.18	7.74	*TIP41*	7.24
	8	*UBC*	1.45	*TIP41*	0.26	*UBC*	3.26	11.54	*eIF-4α*	7.74
GA	1	*TUB*	0.18	*EF-1α*	0.02	*eIF-4α*	0.72	2.88	*EF-1α*	1.19
	2	*GAPDH*	0.18	*GAPDH*	0.02	*ACTIN*	0.82	3.37	*TUB*	2.45
	3	*EF-1α*	0.21	*TUB*	0.03	*TUB*	1.00	4.24	*SAND*	3.13
	3	*TIP41*	0.47	*TIP41*	0.07	*EF-1α*	1.03	4.79	*GAPDH*	3.46
	5	*SAND*	0.72	*UBC*	0.10	*GAPDH*	1.05	4.34	*ACTIN*	3.66
	6	*eIF-4α*	1.21	*eIF-4α*	0.19	*TIP41*	1.60	5.57	*eIF-4α*	5.23
	7	*ACTIN*	1.42	*SAND*	0.23	*SAND*	2.14	7.73	*TIP41*	7.00
	8	*UBC*	1.84	*ACTIN*	0.24	*UBC*	3.76	13.56	*UBC*	8.00
ABA	1	*EF-1α*	0.21	*UBC*	0.01	*GAPDH*	0.58	2.36	*EF-1α*	1.00
	2	*GAPDH*	0.21	*EF-1α*	0.01	*EF-1α*	0.62	2.82	*GAPDH*	1.68
	3	*TUB*	0.32	*GAPDH*	0.03	*eIF-4α*	0.64	2.56	*TUB*	3.00
	3	*TIP41*	0.51	*TUB*	0.03	*ACTIN*	0.80	3.30	*ACTIN*	4.60
	5	*SAND*	0.62	*TIP41*	0.09	*TUB*	0.90	3.81	*eIF-4α*	5.23
	6	*UBC*	0.75	*eIF-4α*	0.09	*SAND*	1.21	4.23	*UBC*	5.63
	7	*eIF-4α*	1.04	*SAND*	0.11	*TIP41*	1.30	4.50	*TIP41*	6.19
	8	*ACTIN*	1.21	*ACTIN*	0.14	*UBC*	1.78	5.99	*SAND*	8.00
MeJA	1	*EF-1α*	0.21	*UBC*	0.04	*ACTIN*	0.27	1.15	*EF-1α*	1.68
	2	*GAPDH*	0.40	*TIP41*	0.07	*eIF-4α*	0.70	2.98	*GAPDH*	2.06
	3	*TUB*	0.44	*EF-1α*	0.08	*GAPDH*	1.57	6.64	*eIF-4α*	2.45
	3	*TIP41*	0.44	*GAPDH*	0.09	*EF-1α*	1.60	7.68	*TUB*	3.83
	5	*SAND*	0.54	*SAND*	0.11	*TUB*	2.01	8.96	*UBC*	4.16
	6	*eIF-4α*	0.78	*TUB*	0.15	*TIP41*	2.08	7.37	*SAND*	4.86
	7	*ACTIN*	1.02	*eIF-4α*	0.20	*SAND*	2.29	8.32	*TIP41*	6.44
	8	*TUB*	1.41	*ACTIN*	0.31	*UBC*	3.99	14.49	*ACTIN*	8.00
SA	1	*EF-1α*	0.29	*EF-1α*	0.02	*ACTIN*	0.20	0.85	*EF-1α*	1.19
	2	*GAPDH*	0.29	*TUB*	0.02	*eIF-4α*	0.35	1.47	*ACTIN*	1.41
	3	*TUB*	0.35	*GAPDH*	0.03	*GAPDH*	0.74	3.02	*TIP41*	3.41
	3	*SAND*	0.46	*SAND*	0.07	*EF-1α*	0.92	4.26	*eIF-4α*	3.94
	5	*eIF-4α*	0.56	*eIF-4α*	0.09	*TUB*	1.12	4.78	*TUB*	4.47
	6	*ACTIN*	0.66	*ACTIN*	0.14	*SAND*	1.14	3.97	*GAPDH*	6.24
	7	*TIP41*	0.83	*TIP41*	0.15	*TIP41*	1.89	6.67	*SAND*	6.96
	8	*UBC*	1.47	*UBC*	0.30	*UBC*	4.18	15.28	*UBC*	7.74

**Table 3:** **Gene expression stability under multiple stress conditions ranked by geNorm,NormFinder, and BestKeeper**.

**Treatments**	**Rank**	**geNorm**		**NormFinder**		**BestKeeper**			**RefFinder**	
		**Gene**	**Stability**	**Gene**	**Stability**	**Gene**	**SD**	**CV**	**Gene**	**Comprehensive ranking values**
Abiotic stress	1	*TUB*	0.48	*EF-1α*	0.07	*ACTIN*	0.62	2.65	*TUB*	1.68
	2	*EF-1α*	0.48	*TUB*	0.10	*TUB*	0.66	2.84	*EF-1α*	1.73
	3	*SAND*	0.60	*SAND*	0.10	*eIF-4α*	0.76	3.24	*TIP41*	2.63
	3	*TIP41*	0.64	*TIP41*	0.17	*GAPDH*	0.77	3.14	*SAND*	2.78
	5	*GAPDH*	0.78	*ACTIN*	0.18	*EF-1α*	0.77	3.68	*ACTIN*	4.73
	6	*ACTIN*	1.00	*eIF-4α*	0.25	*SAND*	1.08	3.91	*eIF-4α*	6.24
	7	*eIF-4α*	1.10	*GAPDH*	0.26	*TIP41*	1.35	4.88	*GAPDH*	6.74
	8	*UBC*	1.53	*UBC*	0.28	*UBC*	3.62	13.48	*UBC*	8.00
Hormone stimuli	1	*EF-1α*	0.21	*EF-1α*	0.05	*EF-1α*	0.84	4.04	*EF-1α*	1.00
	2	*GAPDH*	0.21	*GAPDH*	0.09	*GAPDH*	0.85	3.61	*GAPDH*	2.28
	3	*TUB*	0.35	*TIP41*	0.10	*ACTIN*	0.86	3.57	*TUB*	2.63
	3	*TIP41*	0.54	*TUB*	0.13	*TUB*	0.92	4.07	*TIP41*	3.36
	5	*SAND*	0.70	*eIF-4α*	0.14	*eIF-4α*	1.00	4.08	*ACTIN*	5.00
	6	*eIF-4α*	0.96	*ACTIN*	0.20	*TIP41*	1.21	4.40	*eIF-4α*	6.24
	7	*ACTIN*	1.08	*SAND*	0.21	*SAND*	1.44	5.31	*SAND*	6.74
	8	*UBC*	1.45	*UBC*	0.23	*UBC*	2.96	11.38	*UBC*	8.00
Total	1	*TUB*	0.56	*EF-1α*	0.06	*TUB*	0.68	3.03	*EF-1α*	1.19
	2	*EF-1α*	0.56	*TIP41*	0.14	*EF-1α*	0.68	3.31	*TUB*	2.11
	3	*GAPDH*	0.64	*TUB*	0.15	*ACTIN*	0.72	3.05	*TIP41*	2.28
	3	*TIP41*	0.72	*ACTIN*	0.19	*TIP41*	0.89	3.27	*ACTIN*	4.43
	5	*SAND*	0.76	*SAND*	0.19	*eIF-4α*	0.96	4.02	*SAND*	4.73
	6	*ACTIN*	0.98	*GAPDH*	0.21	*GAPDH*	0.97	4.10	*GAPDH*	5.05
	7	*eIF-4α*	1.09	*eIF-4α*	0.22	*SAND*	1.02	3.80	*eIF-4α*	7.00
	8	*UBC*	1.44	*UBC*	0.27	*UBC*	2.64	10.28	*UBC*	8.00

**Figure 3:**
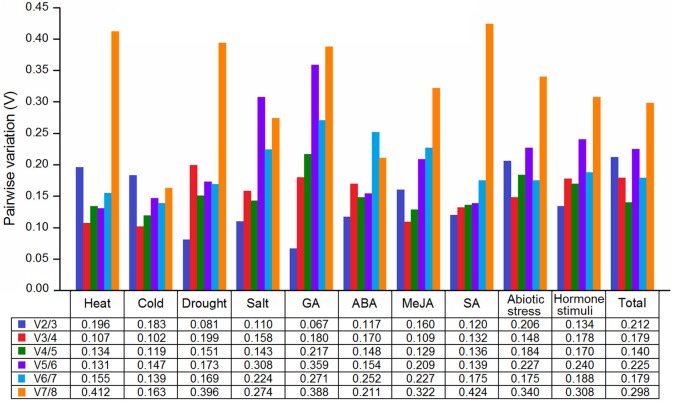
**Determination of the optimal numbers of reference genes in parsley**.

The pairwise variation (V_n/n+1_) was calculated to determine the optimal number of reference genes for various groups. A V_n/n+1_ below 0.15 indicates that introducing an additional reference gene for normalization is not necessary. As shown in Figure 3, except for the cold and heat treatments, the V_2/3_ values were lower than 0.15 under the other six conditions, indicating that the two reference genes were sufficient for normalization; three reference genes (V_3/4_ < 0.15) were proposed to be used in cold and heat conditions. For group “Hormone stimuli,” two reference genes were sufficient for gene normalization with the V_2/3_ value below the threshold value of 0.15. For groups “Abiotic stress” and “Total,” three and four reference genes were required, respectively. Using more number of reference genes may help in reducing system deviation, but it does not result in more reliable results.

#### NormFinder analysis

The calculation principle of NormFinder differed slightly from geNorm. NormFinder ranks the candidate reference genes based on intragroup and intergroup expression variations. A smaller *M*-value indicates that the gene is more stable. As shown in Table 2, *TIP41* and *EF-1*α were the two most stable genes under heat condition, *eIF-4*α and *TUB* under cold and salt conditions, *EF-1*α under salt, SA, and GA treatments, and *UBC* under ABA and MeJA treatments. For the combination analysis (Table 3), *EF-1*α ranked the first in all three groups. Similar to the results generated by geNorm, *EF-1*α and *TUB* displayed good performance, whereas *UBC* was the least stable reference gene.

#### BestKeeper analysis

BestKeeper ranks the reference genes by calculating the SD and the CV of their Cq values, and small SD- and CV-values indicate that the gene is more stable. For the eight individual treatments (Table 2), *ACTIN* had the lowest SD- and CV-values under drought, salt, MeJA, and SA treatments. *eIF-4*α was the most stable gene in cold and GA treatments. For the three groups (Table 3), both *ACTIN* and *TU*B were the most stable genes in “Abiotic stress,” and *EF-1*α and *GAPD*H were the most stable in “Hormone stimuli.” In “Total,” *TUB* and *EF-1*α were more stable than other genes. In addition, we also discovered that *UBC* was the least stable gene in most sets.

#### RefFinder

RefFinder was used to integrate a comprehensive ranking of the most stable candidate genes. However, the ranking orders generated by geNorm, NormFinder, BestKeeper, and RefFinder were not entirely consistent for the eight individual treatments (Table 2). Comprehensive ranking for the three groups revealed that *EF-1*α and *TUB* were the most stable genes in “Abiotic stresses,” while *EF-1*α, *GAPDH*, and *TUB* had good performance in “Hormone stimuli” (Table 3). In “Total,” *EF-1*α and *TUB* can be recommended as the optimal reference genes for normalization in various conditions.

### Validation of the best and least ranked reference genes

The DREB transcription factors are important members of the AP2/ERF family, which play important roles in the regulation of plant stress response (Liu et al., 2000; Agarwal et al., 2006; Zhuang et al., 2008; Wu et al., 2015). In *Arabidopsis*, a number of DREB genes have been induced to be expressed under drought and cold stresses (Seki et al., 2001). Overexpression of a cotton DREB gene, *GhDREB*, can increase drought, salt, and freezing tolerance of transgenic wheat (Gao et al., 2009). To validate the selected reference genes, the expression levels of *PcDREB1* and *PcDREB2* genes were evaluated by qPCR under cold and drought conditions. Four reference genes, including the two most stable genes, *TUB* and *EF-1*α, one less stable gene *eIF-4*α, and the least stable gene *UBC*, were selected to normalize the expression of target genes.

**Figure 4:**
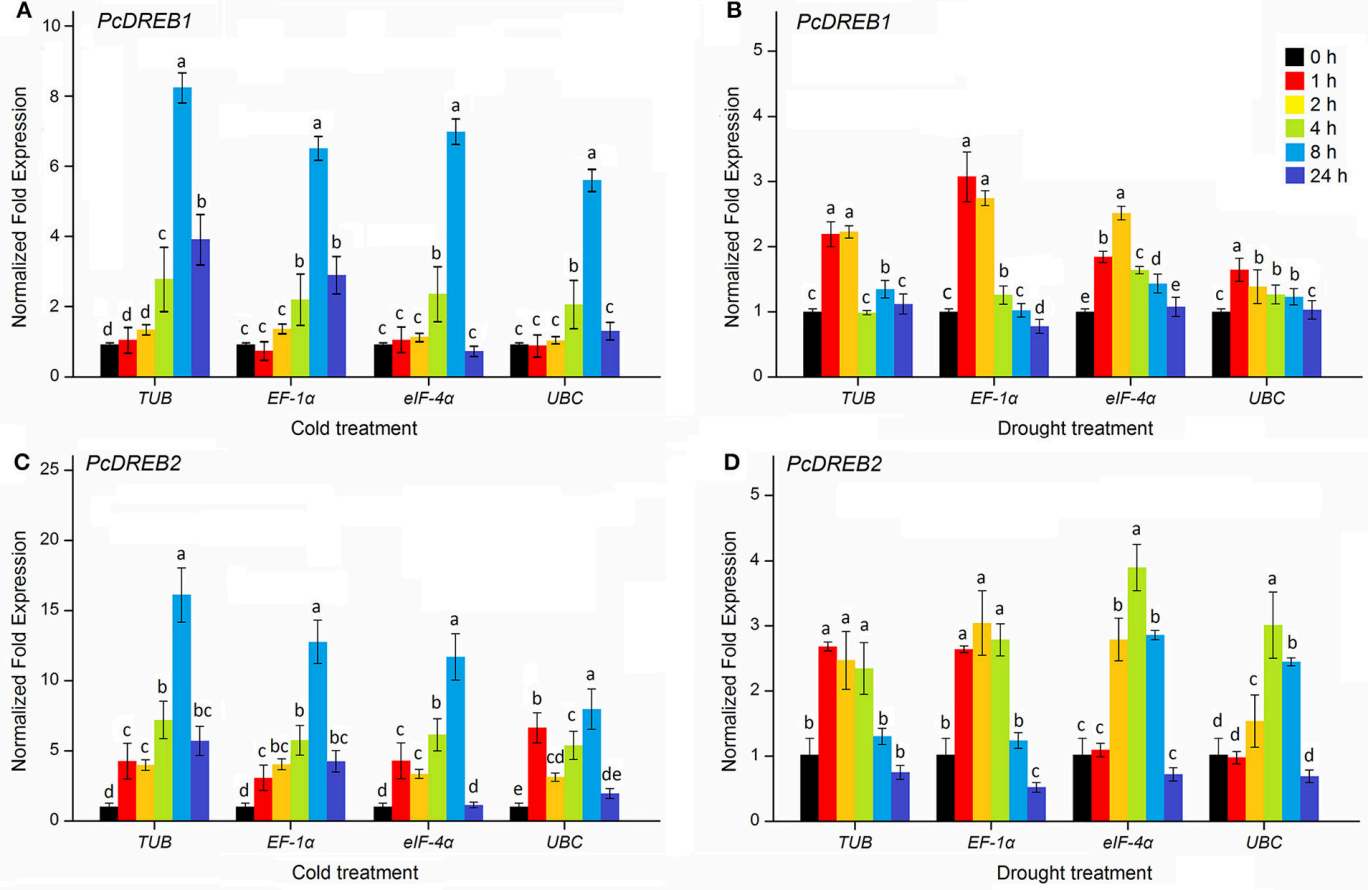
**Relative expression patterns of *PcDREB1* (A,B) and *PcDREB2* (C,D) under cold and drought conditions**. *TUB, EF-1*α, *eIF-4*α, and *UBC* were used as reference genes for expression normalization. Different letters on the vertical bars indicate significant difference at 0.05 levels.

As shown in Figure 4A, under cold condition, *PcDREB1* showed similar response pattern when normalized by the four reference genes (*TUB, EF-1*α*, eIF-4*α, and *UBC*): expression level initially increased, reached the highest level at 8 h, and finally decreased afterward. Meanwhile, the expression levels during normalization with *TUB* and *EF-1*α were maintained at high levels at 24 h. In response to drought treatment, the change in the expression level of *PcDREB1* was different (Figure 4B). Normalization with *TUB* and *EF-1*α indicated a 2- or 3-fold increase after 1 h of drought treatment and maintenance of high levels at 2 h, whereas normalization with *UBC* indicated little change during these stages. Differences in results were also observed in *PcDREB2*. The expression patterns of *PcDREB2* were similar when the most stable genes *TUB* and *EF-1*α were used for normalization, but varied greatly during normalization with the least stable gene *UBC* (Figure 4C). Under drought stress, *PcDREB2* responded quickly to drought, and high expression level was maintained at early stages (1–4 h) when using *TUB* and *EF-1*α as reference genes. On the other hand, expression levels of *PcDREB2* at 4 and 8 h was higher when using e*IF-4*α and *UBC* as reference genes (Figure 4D). Overall, the expression patterns of *PcDREB1* or *PcDREB2* were consistent when using the *TUB* and *EF-1*α, whereas the expression levels demonstrated some deviations when the less stable genes were used. In view of these results, *TUB* and *EF-1*α were considered as the suitable reference genes for normalizing qPCR data.

## Discussion

qPCR is one of the most sensitive methods that can detect the low expression of target genes (Bustin, 2000). In qPCR quantification, reference genes are required for eliminating the errors during mRNA extraction, amplification efficiency, qPCR procedure, and so on (Vanguilder et al., 2008; Derveaux et al., 2010). An ideal reference gene should satisfy the following requirements: stable expression in different tissue or organ, expression is not affected by any experimental conditions, gene is expressed to a certain degree, and the expression level is similar to that of the target gene (Thellin et al., 1999; Suzuki et al., 2000; Udvardi et al., 2008). Some genes, which are involved in cytoskeleton structure (*ACTIN* and *TUB*), protein synthesis (*EF-1*α and *eIF-4*α), and biological metabolic processes (*GAPDH* and *UBQ*), are called housekeeping genes and are usually used as reference genes (Rebouças et al., 2013). To date, with the obtained genome and transcriptome data, some new generation reference genes such as *Fb15* (fiber protein15), *ABCT* (ATP-binding cassette transporter), and *CAC* (clathrin adaptor complexes medium) were shown to have stable expression similar to the traditional reference genes (Xu et al., 2015; Reddy D. S. et al., 2016).

In this study, using parsley, the stability of eight reference genes (*GAPDH, ACTIN, eIF-4*α, *SAND, UBC, TIP41, EF-1*α, and *TUB*) under different abiotic stresses and hormone stimuli was analyzed. Three software, geNorm (Vandesompele et al., 2002), NormFinder (Andersen et al., 2004), BestKeeper (Pfaffl et al., 2004), were used to evaluate the expression stability of candidate reference genes. Results obtained from geNorm, NormFinder, and BestKeeper were not consistent, especially under specific individual conditions. For example, in heat treatment, *GAPDH* was the most stable gene in BestKeeper analysis, yet *GAPDH* preformed unsatisfactorily in geNorm and NormFinder. In cold treatment, geNorm and BestKeeper both recommended *GAPDH* and *eIF-4*α as the suitable reference genes, whereas *TUB* and *eIF-4*α were identified as suitable genes by NormFinder. However, no significant difference was observed in the expression stability between *TUB* and *GAPDH* under cold conditions. Therefore, we inferred that these three genes can be used for the correction of relative gene expression. The divergences among the three software were possibly due to the differences in algorithms. However, synthesizing several multiple results from different software can minimize the errors on the selection of reference genes. Two or more statistical software were used for reference gene selection in previous literature, geNorm, NormFinder, and BestKeeper were the three most commonly used software (Qi et al., 2016; Reddy P. S. et al., 2016). RefFinder (Xie et al., 2012) was used to generate a comprehensive ranking of candidate reference genes. Although *ACTIN* ranked the first in BestKeeper analysis under abiotic stress condition, by comprehensive analysis, RefFinder recommended *TUB* and *EF-1*α as the most stable reference genes. Results from geNorm, NormFinder, BestKeeper, and RefFinder in “Hormone stimuli” were consistent, which showed that *EF-1*α and *GAPDH* were the most stably expressed genes. We also found that *TUB* had good performance in “Hormone stimuli.” By comprehensive comparison, *EF-1*α and *TUB* can be used as the most suitable reference genes in various conditions.

In most previous studies, reference genes were not always stable in different tissues, organs, genotypes, and experimental conditions (Schmittgen and Zakrajsek, 2000). In rice, *UBQ5* and *eEF-1*α are the most stable genes in all the tissue samples, and *18S* and *25S rRNAs* are the most stable genes under various treatment conditions (Jain et al., 2006). The other study showed that *eIF-4*α and *ACT1* performed well at different developmental stages and different varieties of rice, while *18S* and *25S rRNAs* had the least stable expression (Li et al., 2010). Moreover, the selections of reference genes are not the same in various species. Carrot is another important vegetable of the Apiaceae family. Tian et al. (2015) discovered that *ACTIN* and *TUB* in carrot are the most suitable reference genes among “Abiotic stress” and “Hormone stimuli,” but *ACTIN* was not the suitable choice in our study. *GAPDH* showed good performance in “Hormone stimuli” in parsley but was the least stable in carrot. However, *TUB* performed satisfactorily in these two plants. To accurately analyze the expression patterns of the target genes, selecting the proper reference genes according to the species and experimental requirement is necessary.

Plant growth and development are generally affected by abiotic stresses, such as extreme temperature, drought or high-salinity conditions. Increasing evidence have demonstrated that AP2/ERF transcription factors play important roles in plant development and stress response (Xu et al., 2011; Licausi et al., 2013; Zhuang et al., 2014). DREB proteins are members of a class of AP2/ERF transcription factor family that can directly regulate various stress related genes by binding the DNA with the DRE/CRT element, which can increase plant resistance to abiotic stress (Sakuma et al., 2002). The functions of DREB genes have been characterized in many plants. For example, overexpression of *ZmDREB1A* in transgenic plants can increase tolerance of plants to drought and freezing stresses (Qin et al., 2004). Similarly, overexpression of *GmDREB* gene from soybean resulted in enhanced drought and cold stress tolerance in transgenic wheat plants (Gao et al., 2005). In this study, the expression levels of *PcDREB1* and *PcDREB2* were assessed under cold and drought conditions to validate the selected reference genes. When using the two most stable genes *TUB* and *EF-1*α, the expression of the target gene was consistent. *PcDREB1* and *PcDREB2* genes were up-regulated under cold and drought stresses, which were in agreement with other DREB genes responding to cold and drought stresses (Shinwari et al., 1998; Ito et al., 2006). Normalization with the less stable gene *eIF-4*α showed little change in the expression of the gene. In contrast, some divergences were observed in the expression patterns, which normalized by the least stable reference gene *UBC*. The observations were as follows: the expression of *PcDREB1* had no significant change under drought condition, and the expression level of *PcDREB2* under cold stress increased initially, decreased at 2 h, increased again, and finally declined. Results demonstrated that using an unstable reference gene for normalization may contribute to inaccurate results.

In conclusion, we evaluated the stabilities of eight candidate reference genes during treatments with various abiotic stresses (heat, cold, salt, and drought) and hormone stimuli treatments (GA, SA, MeJA, and ABA). Our study proposed that *EF-1*α and *TUB* are the suitable genes under abiotic stresses; *EF-1*α, *GAPDH*, and *TUB* are suitable for normalization during hormone stimuli treatments. Overall, *EF-1*α and *TUB* can be used under all above conditions. In addition, to verify the selected reference genes, the expression patterns of *PcDREB1* and *PcDREB2* were analyzed. The reference genes selected in this study provide more choices in genes expression analysis and functional studies in parsley.

## Author contributions

AX and ML initiated and designed the research. ML, XS, and FW performed the experiments. ML, XS, FW, and AX analyzed the data. AX contributed reagents/materials/analysis tools. ML wrote the paper. ML and AX revised the paper.

### Conflict of interest statement

The authors declare that the research was conducted in the absence of any commercial or financial relationships that could be construed as a potential conflict of interest.

## Supplementary material

Figure S1 **Standard curves of eight candidate reference genes in parsley. (A)** *eIF-4*α; **(B)** *ACTIN*; **(C)** *TIP41*; **(D)** *GAPDH*; **(E)** *SAND*; **(F)** *EF-1*α; **(G)** *TUB*; **(H)** *UBC*.

## Abbreviations

ABA: abscisic acid
*eIF-4*α: Eukaryotic translation initiation factor 4α-1 gene
*EF-1*α: Elongation factor-1α gene
GA: gibberellins
*GAPDH*: Glyceraldehyde-3-phosphate dehydrogenase gene
MeJA: methyl jasmonate
qPCR: quantitative real-time reverse transcription PCR
SA: salicylic acid
*TIP41*: Tap42-interacting protein of 41 kDa gene.
