# Supplementary figures and images for: Suitable Reference Genes for Accurate Gene Expression Analysis in Parsley (Petroselinum crispum) for Abiotic Stresses and Hormone Stimuli
Source: Front Plant Sci. 2016 Sep 30;7:1481. doi: 10.3389/fpls.2016.01481 (PMC5043067; doi:10.3389/fpls.2016.01481)

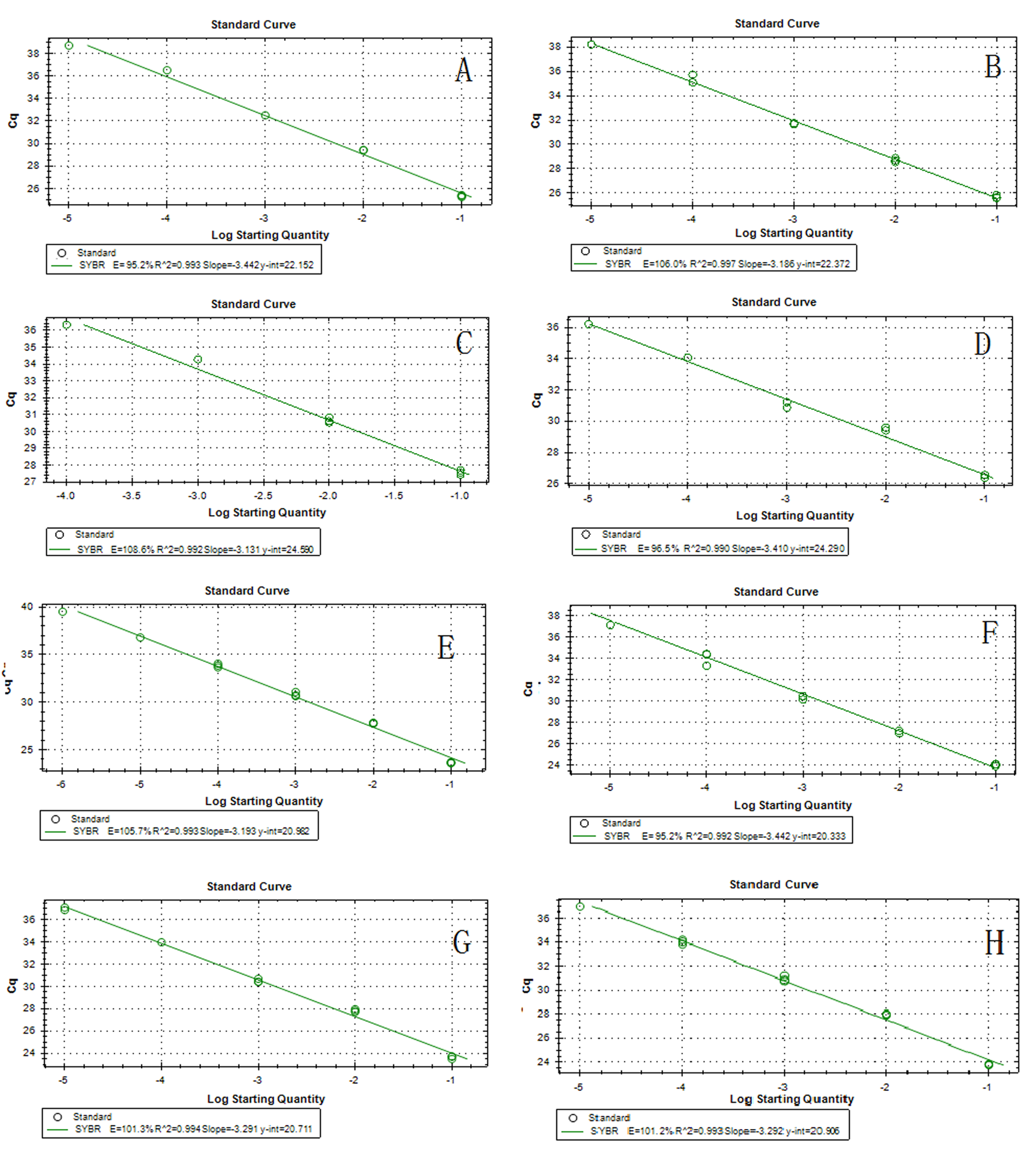

Supplement: Figure S1 — Standard curves of eight candidate reference genes in parsley. (A) eIF-4α; (B) ACTIN; (C) TIP41; (D) GAPDH; (E) SAND; (F) EF-1α; (G) TUB; (H) UBC. [file Image1.TIF]
